# Kindlin-2 suppresses transcription factor GATA4 through interaction with SUV39H1 to attenuate hypertrophy

**DOI:** 10.1038/s41419-019-2121-0

**Published:** 2019-11-26

**Authors:** Lihua Qi, Xiaochun Chi, Xi Zhang, Xueqian Feng, Wenhui Chu, Shengchang Zhang, Junzhou Wu, Yao Song, Youyi Zhang, Wei Kong, Yu Yu, Hongquan Zhang

**Affiliations:** 1 Key Laboratory of Carcinogenesis and Translational Research (Ministry of Education), Department of Human Anatomy, Histology and Embryology, and State Key Laboratory of Natural and Biomimetic Drugs, Peking University Health Science Center, Beijing, China; 2 Anatomy Teaching and Research Section, Guangxi Traditional Chinese Medical University, Guangxi Zhuang Autonomous Region, Nanning, China; 3 Zhejiang Cancer Research Institute, Zhejiang Cancer Hospital, Hangzhou, China; 4 Institute of Vascular Medicine, Peking University Third Hospital and Key Laboratory of Molecular Cardiovascular Sciences (Ministry of Education), Key Laboratory of Cardiovascular Molecular Biology and Regulatory Peptides (Ministry of Health), Beijing, China; 5 Department of Physiology and Pathophysiology, Peking University Health Science Center, Beijing, China

**Keywords:** Mechanisms of disease, Cardiac hypertrophy

## Abstract

Kindlin-2 plays an important role in the regulation of cardiac structure and function. Depletion of Kindlin-2 contributes to cardiac hypertrophy and progressive heart failure, however, the precise mechanisms involved in this process remain unclear*. GATA4* is a critical transcription factor in regulating cardiogenesis. We found that Kindlin-2 suppresses the expression of *GATA4* through binding to its promoter and prevents cardiomyocytes from hypertrophy induced by isoproterenol (ISO) treatment. Mechanistically, Kindlin-2 interacts with histone methyltransferase SUV39H1 and recruits it to *GATA4* promoter leading to the occupancy of histone H3K9 di- and tri-methylation. Furthermore, to confirm the function of Kindlin-2 in vivo, we generated mice with targeted deletion of cardiac Kindlin-2. We found that 6-month-old Kindlin-2 cKO mice have developed hypertrophic cardiomyopathy and that this pathological process can be accelerated by ISO-treatment. *GATA4* expression was markedly activated in cardiac tissues of Kindlin-2 cKO mice compared to wild-type animals. Collectively, our data revealed that Kindlin-2 suppresses *GATA4* expression by triggering histone H3K9 methylation in part and protects heart from pathological hypertrophy.

## Introduction

Hypertrophic cardiomyopathy is characterized by left ventricular hypertrophy accompanied by diastolic dysfunction^1^. During myocardial hypertrophy, various hypertrophic responsive genes are directly activated by transcription factors including *GATA4, AP-1, Sp1, SRF, TEF-1* and *NFAT*^2–4^. *GATA4*, a member of the *GATA* transcription factor family, is highly expressed in the heart and activates transcription of hypertrophic responsive genes including α-myosin heavy chain, cardiac troponin C, atrial natriuretic peptide (*ANP*), and brain natriuretic peptide (*BNP*)^5,6^. Overexpression of *GATA4* induces hypertrophy in both cultured cardiomyocytes and hearts of mice^5^. Deletion of *GATA4* in embryos results in defects of early cardiac development indicating the essential role of *GATA4* in cardiogenesis^6,7^. *GATA4* expression can be modulated by epigenetic modifications, including DNA methylation and histone acetylation. However, the regulation of histone methylation on *GATA4* expression is still poorly understood.

Integrins are transmembrane adhesion receptors which regulate bidirectional signaling across the cell membrane^8^. Integrins, together with integrin-interacting proteins, play critical roles in normal cardiac muscle function^9–11^. Cardiac myocyte-specific loss of the beta1 integrin results in myocardial fibrosis and dilated cardiomyopathy^10^. Targeted deletion of integrin-linked kinase in murine heart leads to dilated cardiomyopathy and spontaneous heart failure^11^. Kindlin-2, as an integrin-interacting protein, activates integrin by binding to the cytoplasmic tail of integrin beta and mediates cell–cell and cell–matrix adhesion^12^. Knockdown of Kindlin-2 in zebrafish contributes to severe abnormalities in cardiac structure and function^13^. Further, Kindlin-2 plays a role in maintaining the integrity of Z disc in postnatal mice and knockdown of Kindlin-2 disrupts Z-disc structures resulting in cardiac dysfunction^14^. Targeted deletion of Kindlin-2 in murine heart leads to cardiomyopathy and progressive heart failure by decreasing the level of integrin beta^15^. However, whether Kindlin-2 directly regulates cardiac-specific transcription factors remains unknown.

In this study, we found that Kindlin-2 negatively regulates hypertrophic transcription factor *GATA4* by directly binding to the promoter of *GATA4* in neonatal cardiomyocytes. Kindlin-2 has been reported to interact with DNA methyltransferases 1 and 3a to suppress gene expression^16,17^. Here, we found that Kindlin-2 interacts with histone methyltransferase supperssor of variegation3-9 homolog1 (SUV39H1) and recruits it to *GATA4* promoter, leading to the enrichment of the di- and tri-methylation of H3K9, which in turn contributes to silencing of *GATA4*. Further, targeted deletion of Kindlin-2 in heart tissue of mice induced cardiac hypertrophy and that this pathological process can be accelerated by ISO-treatment. Notably, *GATA4* is remarkably activated in mice of Kindlin-2 knockout mice, providing a precise explanation as to Kindlin-2 regulates cardiac hypertrophy.

## Materials and methods

### Animal models

Kindlin-2 floxed C57BL/6 J mice (Kindlin-2^fl/fl^) were generated in our laboratory based on the KO-first Kindlin-2 mice purchased from Europe Mutant Mouse Archive (Germany). To generate cardiac muscle (CM)-specific Kindlin-2 KO mice, Kindlin-2 floxed C57BL/6 J mice were crossed with the same strain mice expressing recombinase alpha (α)-myosin heavy chain (MHC)-Cre. Genotyping of mice was performed by polymerase chain reaction (PCR) analysis using mouse tail DNA and Kindlin-2 primers (forward: 5′-TGTGTTTCAAAGGTACTGGTCA-3′; reverse: 5′-ACAATGGTGCTTTG CCTACA-3′), and Cre primers (forward: 5′-TGTGTTTCAAAGGTAC TGGTCA-3′; reverse, 5′-ACAATGGTGCTTTGCCTACA-3′). We induced cardiac muscle specific Kindlin-2 KO mice using tamoxifen. Briefly, 4-Hydroxytamoxifen (Sigma-Aldrich, St. Louis, MO, USA) was dissolved in corn oil (Sigma) at a concentration of 10 mg/ml. Randomly choice adult (8-10 weeks old) Kindlin-2^fl/fl^ and Kindlin-2^fl/fl^ α-MHC-Cre male mice were given in traperitoneal injections of 4-hydroxytamoxifenonce daily for 1 week at a dose of 30 mg/kg/day. As a control, corn oil alone was injected in the same way. Pathological cardiac hypertrophy was investigated using the isoproterenol-induced subacute myocardial injury model. Briefly, isoproterenol (ISO, Sigma-Aldrich, St. Louis, MO) dissolved in 0.9% saline was injected in abdominal subcutaneous tissue at 10 mg/kg/d. After7 days of ISO administration, mice were sacrificed and processed for subsequent assays. All mice were given saline or ISO twice daily for a period of one week (*n* = 10 for each group). Establishment of hypertrophy and attenuation by ISO was assessed using echo-cardiography by measuring left ventricular (LV) wall thickness and dimensions end-diastole and end-systole. The Ethics Committee of Peking University Health Science Center has approved the mouse experiments (Permit Number: LA2014118) for this study. The handling of mice was conducted in accordance with the ethical standards of the Helsinki Declaration of 1975 and the revised version in 1983.

### RNA isolation, library preparation and sequencing

WT and cKO heart tissues were snap-frozen in liquid nitrogen immediately and then stored at −80 °C before RNA extraction. Total RNA was extracted with Trizol (Tiangen, Beijing) and then assessed with Agilent 2100 BioAnalyzer (Agilent Technologies, Santa Clara, CA, USA) and Qubit Fluorometer (Invitrogen). All of RNA samples that meet the following requirements were used in subsequent experiments: RNA integrity number (RIN) > 7.0 and a 28 S:18 S ratio > 1.8. RNA-seq libraries were generated and sequenced by CapitalBio Technology (Beijing, China). NEB Next Ultra RNA Library Prep Kit for Illumina (NEB) was used to construct the libraries for sequencing. NEB Next Poly(A) mRNA Magnetic Isolation Module (NEB) kit was used to enrich the poly(A) tailed mRNA molecules from 1 μg total RNA. The final libraries were quantified using KAPA Library Quantification kit (KAPA Biosystems, South Africa) and an Agilent 2100 Bioanalyzer. After RT-qPCR validation, libraries were subjected to paired-end sequencing with pair end 150-base pair reading length on an Illumina HiSeq sequencer (Illumina).

### Cell culture and adenovirus infection

Primary rat neonatal cardiomyocytes were isolated from the hearts of 1–3 day old Sprague–Dawley rats. Ventricles were cut into pieces and underwent a series of enzymic digestions with collagenase type II and trypsin at 100 unit/ml and 1 mg/ml, respectively. Cells were re-suspended in Dulbecco’s Modified Eagle’s Medium (DMEM) containing 10% fetal bovine serum and cultured at 37 °C for 120 min to allow fibroblast adhesion. After that, non-adherent cells were collected and then replaced with DMEM medium supplemented with 15% fetal bovine serum and 0.1 mM Brdu. After 24 h, the rat neonatal cardiomyocytes began to beat and then recombinant adenovirus for control and Kindlin-2 infected primary rat neonatal cardiomyocytes at a multiplicity of infection (MOI) with Enhanced Infection Solution (Gene Chem Co, Shanghai, China). After infection for 48 h, cells were treated with saline or 5μm ISO for 24 h. Adenovirus for control and Kindlin-2 were constructed and amplified by Gene Chem Co. (Shanghai, China). Sequence of Kindlin-2 target oligonucleotide was as follows: siRNA-1: AAGUUGGUGGAAAAACUCGAU; siRNA-2: UAUAAGACACCCUGAAGAA.

### Purification of fusion proteins and GST pull-down assays

GST fusion proteins were expressed in *Escherichia coli* strain BL21 (Tiangen Biotechnology, Beijing, China) and purified using glutathione Sepharose 4B beads (Pharmacia Biotech; Pfizer, New York, NY, USA). For GST pull-down assays, GST fusion protein was incubated with glutathione Sepharose 4B beads at 4 °C for 1 h with rocking. The beads were then washed three times with TEN buffer (20 mMTris-HCl, pH 7.4, 0.1 mM EDTA, and 100 mM NaCl). Kindlin-2 antibody was then added to the beads and incubated at 4 °C overnight with rocking. The beads were then washed three times with TENT buffer (0.5% NP40, 20 mMTris-HCl, pH 7.4, 0.1 mM EDTA, and 300 mMNaCl), centrifuged at 3000 × *g* for 1 min, and dissolved in 2 × SDS loading buffer. The solutions were then boiled for 5 min at 100 °C and centrifuged at 12,000 × *g* for 1 min. Finally, the supernatants were removed and analyzed via western blotting.

### Subcellular fraction

Primary rat neonatal cardiomyocytes were rinsed twice in cold PBS, and then incubated with buffer A (50 mM Tris–HCl pH 7.8, 420 mM NaCl, 1 mM EDTA, 0.5% NP40, 0.34 M sucrose, 10% glycerol, 1 mM Na_3_VO_4_, and protease inhibitor mixture for 5 min on ice. After the cells were scraped and centrifuged, the supernatant was the cytoplasmic fraction. Then the pellet was lysed in buffer B (10 mM HEPES, pH 7.9, 10 mM KCl, 1.5 mM MgCl_2_, 0.34 M sucrose, 10% glycerol, 0.1% Triton X-100, protease inhibitor mixture). After centrifuging, the supernatant was the nuclear fraction.

### Co-immunoprecipitation and Western blotting assays

Co-immunoprecipitation (Co-IP) was performed according to a previously described method^18^. Briefly, mouse cardiac muscle or primary rat neonatal cardiomyocytes lysates were incubated with anti-Kindlin-2 (Millipore, Billerics, MA, USA MAB2617, clone 3A3) and anti-SUV39H1 (Cell Signaling Technology, Danvers, MA, USA) antibodies at 4 °C overnight followed by incubation with protein A/G agarose beads (Santa Cruz Biotechonology). After that, the beads were washed three times with NP40 buffer, the bound proteins were eluted with 2 × SDS loading buffer and then boiled at 100 °C for 5 min. Precipitated proteins were resolved by 10% SDS–PAGE and subjected to western blotting analysis. Western blotting were performed by using anti-SUV39H1 (Cell Signaling Technology, Danvers, MA, USA), anti-Kindlin-2 (Millipore, Billerica, MA, USA) antibodies, anti-GATA4 (G-4, Santa Cruz Biotechnology), anti-GATA5 (55433-1-AP, Santa Cruz Biotechnology), anti-GATA6 (YT1885, ImmunoWay Biotechnology) and anti-GAPDH (Santa Cruz Biotechnology). Secondary antibodies were goat anti-mouse HRP and goat anti-rabbit HRP (both Santa Cruz Biotechnology, Inc.).

### Histology

Hearts were fixed with 4% paraformaldehyde overnight at 4 °C and embedded in paraffin. Serial sections (6μm) were stained with hematoxylin and eosin (HE) for histopathological analysis to assess hypertrophy. In order to detect the cell size, the sections were stained with wheat germ agglutinin (WGA) (Sigma) at a concentration of 2 μM at 4 °C overnight then incubated with DAPI for 2 min for the detection of the nuclei. The cell membrane was detected as green fluorescence (488 nm) by confocal microscopy (Leica, Germany). The average diameter of cardiomyocytes was analyzed with image analysis software. In order to detective the expression of GATA4 and Kindlin-2, serial sections (6 μm) were subjected to immunohistochemical staining with ant- Kindlin-2 (Millipore MAB2617, clone 3A3) and anti-GATA4 (G-4 Santa Cruz Biotechnology). All measurements were averaged from three slices. All data were expressed as mean ± S.D. Statistical analysis was performed using Student’s *t*-test. A probability of <0.05 was considered to be statistically significant.

### Chromatin immunoprecipitation

Chromatin immunoprecipitation (ChIP) assays were performed using a ChIP Plus Enzymatic Chromatin IP Kit (NO.9003, Cell Signaling Technology) according to the manufacturer’s instructions. Immunopurification of soluble chromatin was performed using anti-Kindlin-2 (Millipore MAB2617, clone 3A3), anti-SUV39H1 (Cell Signaling Technology, Danvers, MA, USA), anti-H3K9me2 (Cell Signaling Technology, Danvers, MA, USA) and anti-H3K9me3 (Cell Signaling Technology, Danvers, MA, USA) antibodies. The q-ChIP primers: mouse GATA4 (A) forward primer, 5′-AGAGCGCTTGCTCTCG-3′ and reverse primer, 5′- TCCTTGCGGTTTGCTG-3′; GATA4 (B) forward primer, 5′-GCATGGACTTTGCCTG-3′ and reverse primer, 5′-CCTGCGCTGACTGGCCTAAG-3′; GATA4 (C) forward primer, 5′- CGGGAGCAGGGGACAA -3′ and reverse primer, 5′- GCAAACAGGACGGATTA-3′; human GATA4 (B) forward primer 5’-CGACACCCCAATCTCGATATG-3’and reverse primer, 5′-GTTGCACAGATAGTGACCCGT -3′; GATA6 forward primer, 5′- GTTCTTCTCGCACATCGC -3′ and reverse primer, 5′- TGCCCACTGGACTACGG-3′.

### Quantitative real-time PCR (RT-qPCR)

Total RNA was extracted using TRIzol reagent (Invitrogen). cDNA was synthesized using a SuperScript Kit (Invitrogen). The primer sequences used for PCR were; GAPDH forward 5’-CTGAGAACGGGAAGCTTGT-3’ and reverse 5’-GGGTGCTAAGCAGTTGGT-3’; rat GATA4 forward 5’-CCTGCTCTGACTGGCCTAAG -3’and reverse 5’- GCATGGACTTTG CCTGCT-3’; mouse GATA4 forward 5’-GCAGCAGCAGCAGCAGTGAA-3’and reverse 5’-TCTGAGTGACAGGAGATGGATAGCC-3’.

### Luciferase reporter assays

The reporter construct rat GATA4 -pGL3/Luc, ratGATA4 -pGL3/Luc (Mutant 1: -88C allele to -88A allele) and rat GATA4 -pGL3 (Mutant2: -87G allele to -87T) were kindly provided by Dr. Ivana L. de la Serna^19^. For luciferase assays, HEK293Acells were seeded into 24-well plates the day before. Control or Kindlin-2 siRNA was transfected into HEK293Acells with lipofectamine RNAi MAX (Invitrogen). Meanwhile, Flag or Flag-Kindlin-2 was transfected into HEK293Acells with lipofectamine 2000 (Invitrogen). Then, 100 ng of either wild-type GATA4 or mutated GATA4 reporter plasmid with 1 ng of pRL were transfected per well. At 24 h post-transfection, cells were treated with saline or 5 μm ISO for 24 h. Then the reporter activity was measured using a Dual-Luciferase Reporter Assay System (Promega trading company, USA).

### Immunofluorescent staining

After primary rat neonatal cardiomyocytes were fixed with 4% paraformaldehyde solution at room temperature for 15 min, they were treated with 0.5% Triton X-100 at 37 °C for 5 min and blocked with 5% BSA at room temperature for 1 h. The cells were then incubated with 1:200 dilution of anti-Kindlin-2 (Millipore) and dilution of 1:200 anti-SUV39H1 (Cell Signaling Technology) and then with a 1:100 dilution of Alexa Fluor 568-conjugated IgG (Invitrogen) or with a 1:100 dilution of Alexa Fluor 488-conjugated IgG (Invitrogen) for 1 h at room temperature and incubated with DAPI for 2 min for the detection of the nuclei. Images were captured with a TCS SP5 confocal microscope (Leica, Germany).

### Echocardiographic analysis

Echocardiography images were obtained by use of a Veov 770TM Imaging System (Visual Sonics Ini, Toronto, ON, Canada)^20^. Briefly, mice were anesthetized with 1.5–2.0% isoflurane (Baxter Healthcare Corp, New Providence, RI, USA) and maintained on a heating pad to maintain the body temperature within a narrow range (37.0 ± 0.5 °C). Caution was taken not to apply excessive pressure over the chest as this may distort the signals. Two-dimensional parasternal short axis imaging at the level of the papillary muscle was used as a guide to obtain a LV M-mode tracing. Left ventricular internal diameter (Diastole, LVIDd) and left ventricular shortening fraction (%FS) were calculated. All measurements were averaged from three consecutive cardiac cycles. All data were analyzed off-line at the end of the study with software resident on the ultrasound system. All parameters were expressed as mean ± S.D. *n* = 6. Statistical analysis was performed using Student’s *t*-test. A probability of < 0.05 was considered to be statistically significant.

### Statistical analysis

Statistical significance was determined by the two-tailed Student *t* test. *P* < 0.05 was considered statistically significant.

## Results

### Kindlin-2 suppresses expression of hypertrophic transcription factor GATA4

**Fig. 1:**
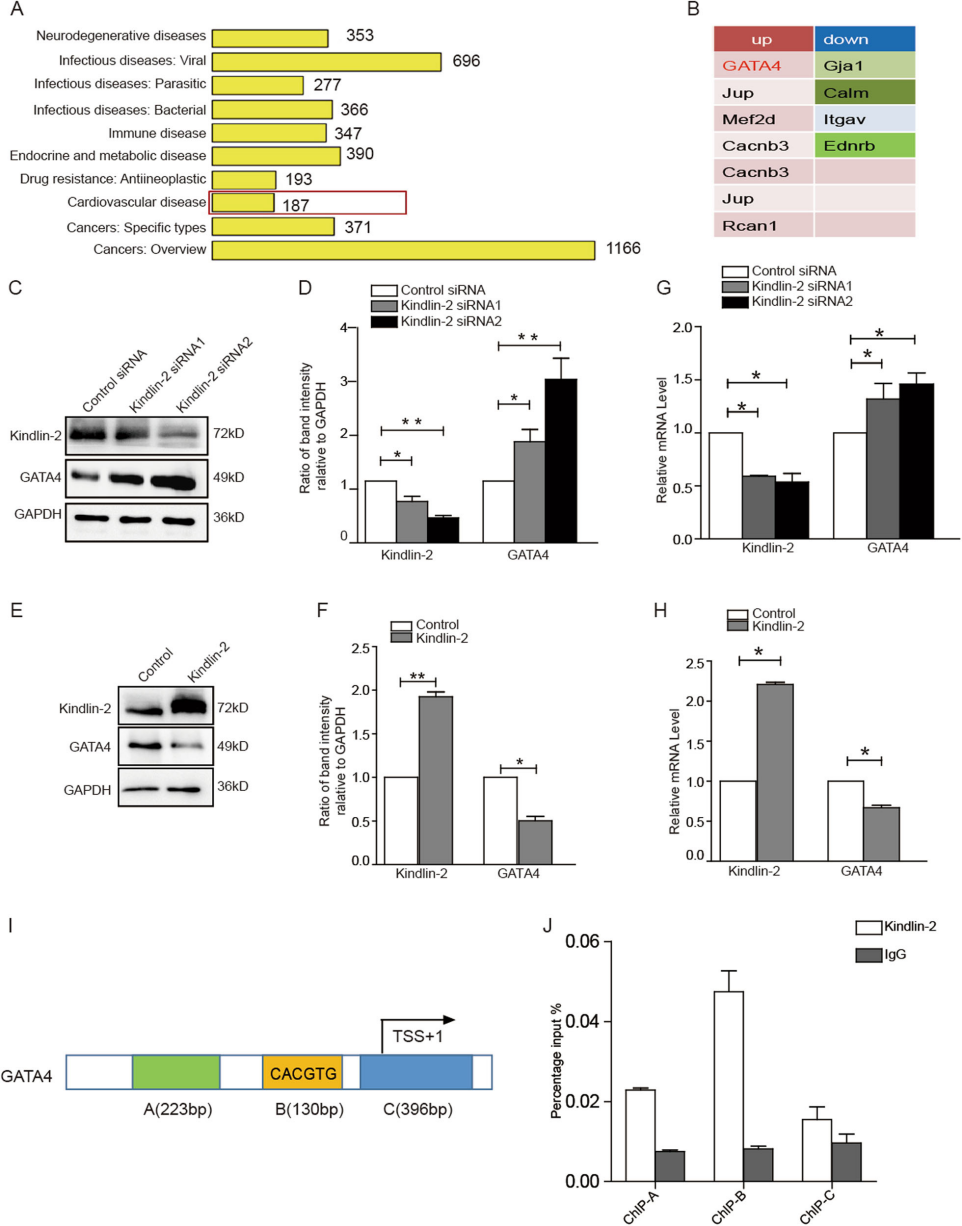
Kindlin-2 suppresses expression of GATA4. **a** GO analysis of differentially expressed genes in RNAseq. **b** 11 differentially expressed genes (log2 Fold > 1) in cardiovascular disease-related genes. **c** Control or Kindlin-2 siRNA was transfected into primary neonate rat cardiomyocytes for 48 h, followed western blot. **d** Protein bands were scanned and relative band intensities were normalized to each GAPDH band. The column diagrams represent average relative band intensity with standard error from three independent experiments. **e** Cardiomyocytes were infected by Kindlin-2 or control adenovirus, followed western blot. **f** Relative band intensities were analyzed. **g** Control or Kindlin-2 siRNA was transfected into cardiomyocytes and RT-qPCR detected Kindlin-2 and *GATA4* mRNA level. **h** Adenovirus carrying Kindlin-2 or control was transfected into cardiomyocytes, followed RT-qPCR. **i** Schematic diagrams of the regions for ChIP assay. **j** Lysates were extracted from mouse cardiac tissues for ChIP assays using anti-Kindlin-2 antibody. Q-PCR assay was then performed to quantify ChIP-enriched DNA using the three primers.

To explore the molecular mechanism underlying Kindlin-2 regulation on cardiac function, we examined the gene expression profiles using control or Kindlin-2 siRNA-treated primary neonate rat cardiomyocytes. GO analysis showed that knockdown of Kindlin-2 influenced the expression of some cardiovascular disease-related genes (Fig. 1a). Among those genes, 7 genes were up-regulated and 4 genes were down-regulated (Fig. 1b). Cardiac-specific transcription factor *GATA4* was noted to be up-regulated by approximately 4.5-fold in Kindlin-2-depleted cardiomyocytes, compared with the control cells (Table. S1). Given to the critical role of *GATA4* in cardiac hypertrophy, we further examined the regulation of Kindlin-2 on *GATA4*. As we expected, knockdown of Kindlin-2 led to an increase in levels of *GATA4* protein in primary neonate rat cardiomyocytes (Fig. 1c, d), whereas overexpression of Kindlin-2 obviously inhibited the protein level of *GATA4* (Fig. 1e, f). Similarly, depletion of Kindlin-2 increased the mRNA levels of *GATA4* (Fig. 1g), whereas overexpression of Kindlin-2 led to reduction of *GATA4* mRNA (Fig. 1h), indicating that Kindlin-2 suppressed the transcription of *GATA4*. Further, we found that Kindlin-2 only affected *GATA4* level in the nucleus (Fig. S1). ChIP assays indicated that Kindlin-2 was markedly occupied on the region B of *GATA4* promoter which included a conserved E box (Fig. 1i, j and Fig. S2A-B). We then cloned the conserved E box of GATA4 into the upstream of firefly luciferase coding region and examined the luciferase activity. Results indicated that knockdown of Kindlin-2 directly activated *GATA4* promoter transcription (Fig. S3A). All these data demonstrated that Kindlin-2 suppressed *GATA4* transcription through binding to its promoter.

### Histone methyltransferase SUV39H1 mediated suppression of Kindlin-2 on GATA4

**Fig. 2:**
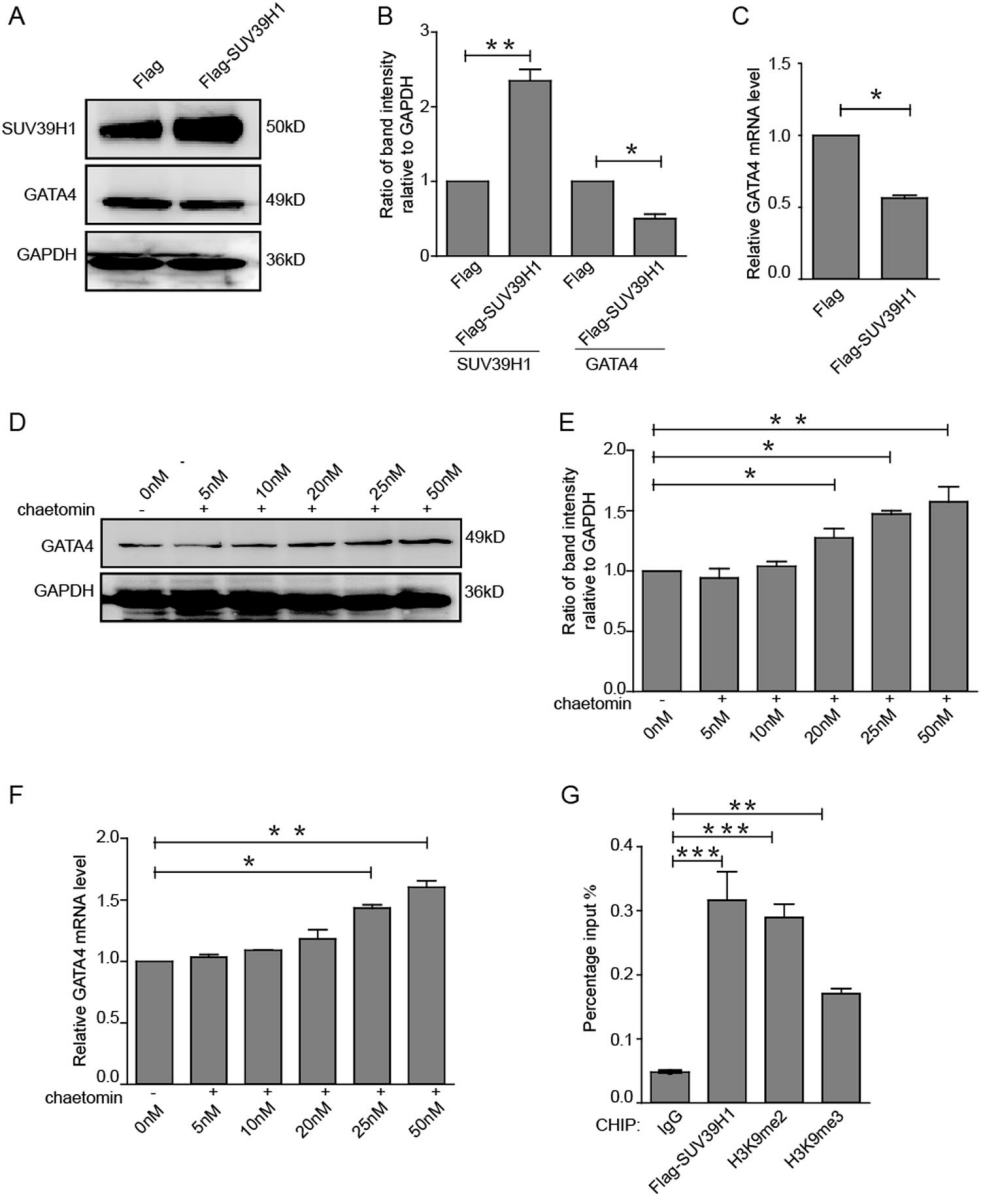
SUV39H1 mediated suppression of Kindlin-2 on GATA4. **a** Flag or Flag-SUV39H1 was transfected into HEK293A cells and *GATA4* were measured by Western blot. **b** Relative band intensities of western blot were analyzed. **c** Flag or Flag-SUV39H1 was transfected into HEK293A cells and *GATA4* were measured by RT-qPCR. **d** HEK293A cells were treated with the SUV39H1 inhibitor Chaetomin at various doses for 48 h, and *GATA4* was measured by western blot. **e** Relative band intensities of western blot were analyzed. **f** HEK293A cells were treated with Chaetomin and GATA4 was detected by RT-PCR. **g** Flag-SUV39H1 was transfected into HEK293A cells and lysates were then extracted for ChIP assays. Means ± S.D. ** indicates *p* < 0.01, *** indicates *p* < 0.001.

Kindlin-2 was reported to function by recruiting various proteins, especially epigenetic regulators^16,17^. SUV39H1 mainly catalyzes the di- and tri-methylation of histone 3 lysine 9 (H3K9me2 and H3K9me3), which suppresses gene expression^21,22^. To explore whether SUV39H1 is involved in the suppression of Kindlin-2 on GATA4, we firstly detected the effect of SUV39H1 on *GATA4* expression. Result showed that overexpression of SUV39H1 led to a reduction in *GATA4* expression both at protein and mRNA levels (Fig. 2a–c). Furthermore, Chaetomin, an inhibitor of SUV39H1 methyltransferase, was used to inhibit the activity of SUV39H1. We found that chaetomin upregulated *GATA4* level (Fig. 2d–f), indicating that SUV39H1 did indeed inhibit *GATA4* expression. Next, luciferase activity of GATA4 promoter was examined upon SUV39H1 overexpression or inhibition by Chaetomin, indicating that SUV39H1 directly inhibited *GATA4* promoter transcription (Fig. S3B). Further, ChIP assays were performed in HEK293Acells overexpressing SUV39H1. We found that SUV39H1 and SUV39H1-catalyzed H3K9me2 and H3K9me3 were occupied on the promoter of *GATA4* (Fig. 2g), suggesting that SUV39H1-mediated H3K9me2 and H3K9me3 are involved in the suppression of *GATA4*.

**Fig. 3:**
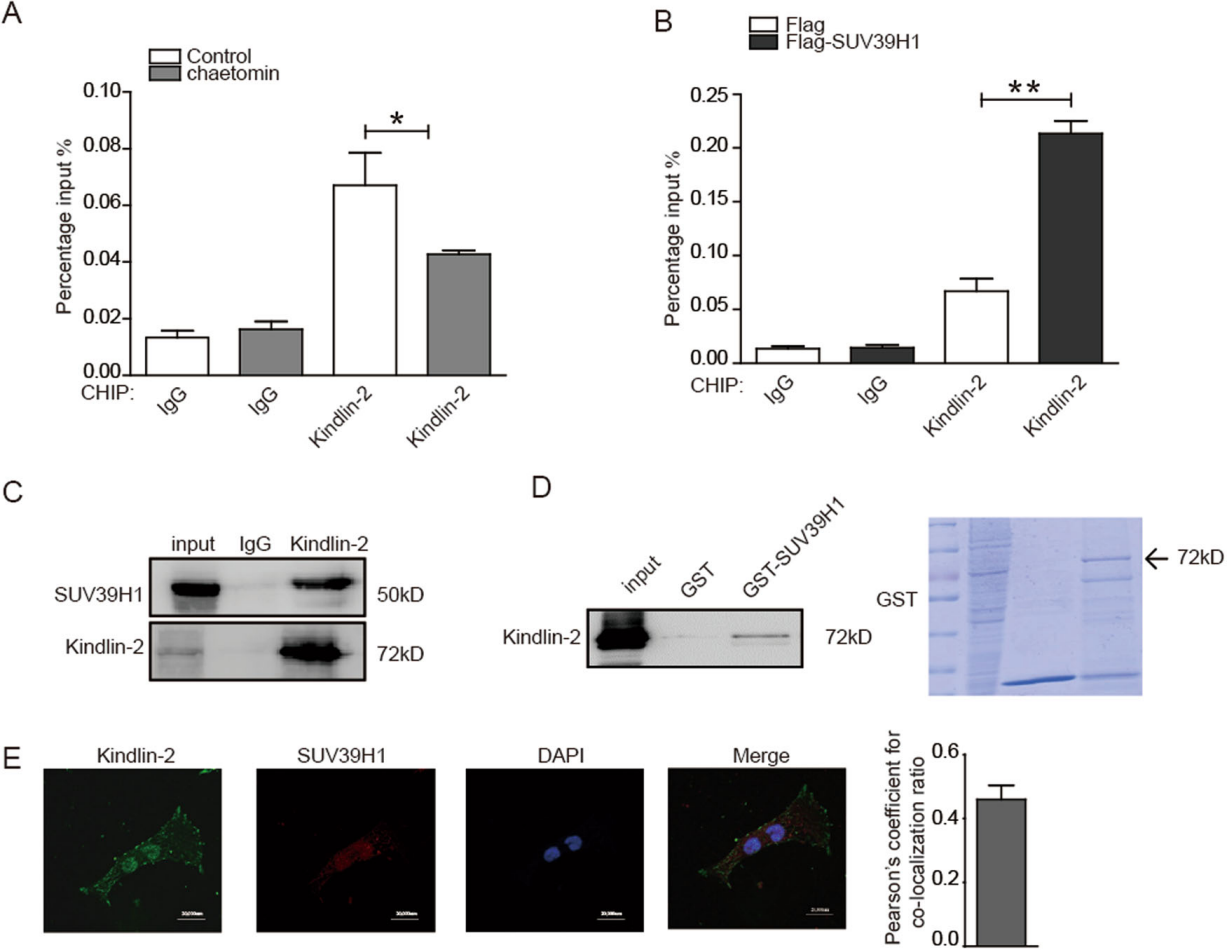
Kindlin-2 interacts with SUV39H1. **a** HEK293A cells were treated by Chaetomin and lysates were then extracted for ChIP assays. **b** Flag-SUV39H1 was transfected into HEK293A cells and lysates were then extracted for ChIP assays. **c** Lysates from HEK293A cells were prepared and then anti-Kindlin-2 antibody was used for Co-IP. **d** Purified GST or GST-SUV39H1 was incubated with total lysates prepared from HEK293A cells for the GST-pull down assays. **e** Immunofluorescent staining of anti-Kindlin-2 (Alexa Fluor 488, green), anti-SUV39H1 (Alexa Fluor 568, red), and nuclei (DAPI, blue) was performed using primary neonate rat cardiomyocytes. Images were captured with a confocal microscope (Scale bar = 20 µm). Pearson’s coefficient was shown in the histogram.

Given to the presence of Kindlin-2 at GATA4 promoter, we speculated whether Kindlin-2 and SUV39H1 work together at GATA4 promoter. We found that inhibition of SUV39H1 by Chaetomin resulted in a marked decrease of Kindlin-2 enrichment at GATA4 promoter (Fig. 3a), whereas overexpression of SUV39H1 led to increased enrichment of Kindlin-2 (Fig. 3b). Further, we analyzed the associations between Kindlin-2 and SUV39H1 and found that Kindlin-2 formed a complex with SUV39H1 in HEK293Acells (Fig. 3c). For GST pull-down assay, purified GST-SUV39H1 was incubated with lysates from HEK293A cells. Results confirmed that GST-SUV39H1 interacted with Kindlin-2 in vitro (Fig. 3d). Immunofluorescent staining in cardiomyocytes revealed that Kindlin-2 and SUV39H1 co-localized in the nucleus (Fig. 3e). These results suggested that Kindlin-2 recruited SUV39H1 to the *GATA4* promoter and in turn induced the enrichments of H3K9me2 and H3K9me3 leading to the suppression of *GATA4*.

### Kindlin-2 prevents cardiomyocytes from hypertrophy through suppressing GATA4

**Fig. 4:**
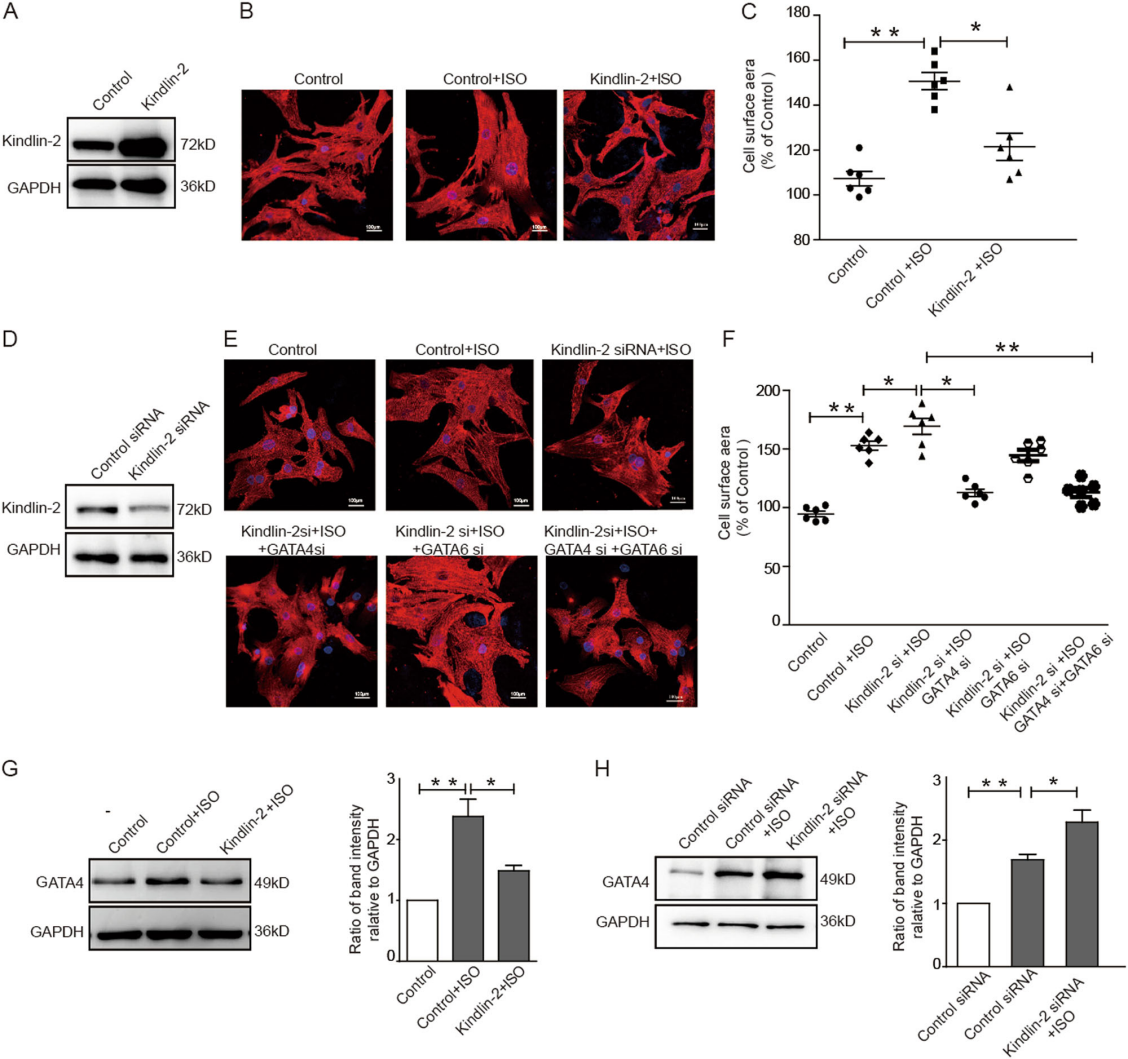
Kindlin-2 prevents cardiomyocytes from hypertrophy through suppressing GATA4. **a–c** Cardiomyocytes were infected with Kindlin-2 or control adenovirus plus ISO treatment (5 µm, 24 h). The efficacy of Kindlin-2 overexpression was detected by Western blot **a**. Representative images of cardiomyocytes stained with anti-α-actinin2 (Alexa Fluor 568) **b**. Cell surface areas were measured **c**. **d**–**f** Kindlin-2 siRNA was transfected into primary neonate rat cardiomyocytes plus ISO treatment with or without knockdown of GATA4 and GATA6 alone or in combination. The efficacy of Kindlin-2 siRNA was detected by Western blotting **d**. Representative images of cardiomyocytes stained with anti-α-actinin2 **e**. Cell surface areas were measured **f**. **g** Cardiomyocytes were infected with Kindlin-2 or control adenovirus plus ISO, followed by Western blot (left panel). Relative band intensities were analyzed (right panel). **h** Cardiomyocytes were transfected with control or Kindlin-2 siRNA plus ISO treatment, followed Western blot (left panel). Relative band intensities were analyzed (right panel).

To explore whether Kindlin-2 regulates the hypertrophy of cardiomyocytes through GATA4, ISO was used to treat primary neonate rat cardiomyocytes to establish a model of cardiomyocyte hypertrophy. Results showed that ISO treatment obviously enlarged the surface area of cardiomyocytes. However, overexpression of Kindlin-2 decreased the surface area of cardiomyocytes (Fig. 4a–c). In contrast, depletion of Kindlin-2 increased the surface area of ISO-treated cardiomyocytes (Fig. 4d–f), suggesting that Kindlin-2 prevents cardiomyocytes from hypertrophy induced by ISO treatment. Further, depletion of Kindlin-2-induced hypertrophic response can be blunted by concomitant knockdown of GATA4, but not GATA6 (Fig. 4d–f), suggesting that GATA4 may be involved in the regulation of Kindlin-2 on cardiomyocyte hypertrophy. Next, ISO treatment led to increased GATA4 level in primary neonate rat cardiomyocytes. Overexpression of Kindlin-2 overcame the activation of GATA4 resulting from ISO treatment (Fig. 4g), whereas depletion of Kindlin-2 further enhanced *GATA4* expression (Fig. 4h). Furthermore, we examined the regulation of Kindlin-2 on the *GATA4* promoter in ISO-treated hypertrophic cardiomyocytes. Results showed that overexpression of Kindlin-2 impaired the activation of wild-type *GATA4* promoter induced by ISO treatment (Fig. S3C), whereas knocking down Kindlin-2 further promoted the activation of wild-type *GATA4* in ISO-treated hypertrophic cells (Fig. S3D). However, the luciferase activity of mutant *GATA4* cannot be regulated by Kindlin-2. These results suggested that Kindlin-2 may be involved in the development of cardiomyocyte hypertrophy by targeting to hypertrophic transcription factor *GATA4*.

### Cardiac-specific Kindlin-2 knockout leads to hypertrophic cardiomyopathy

**Fig. 5:**
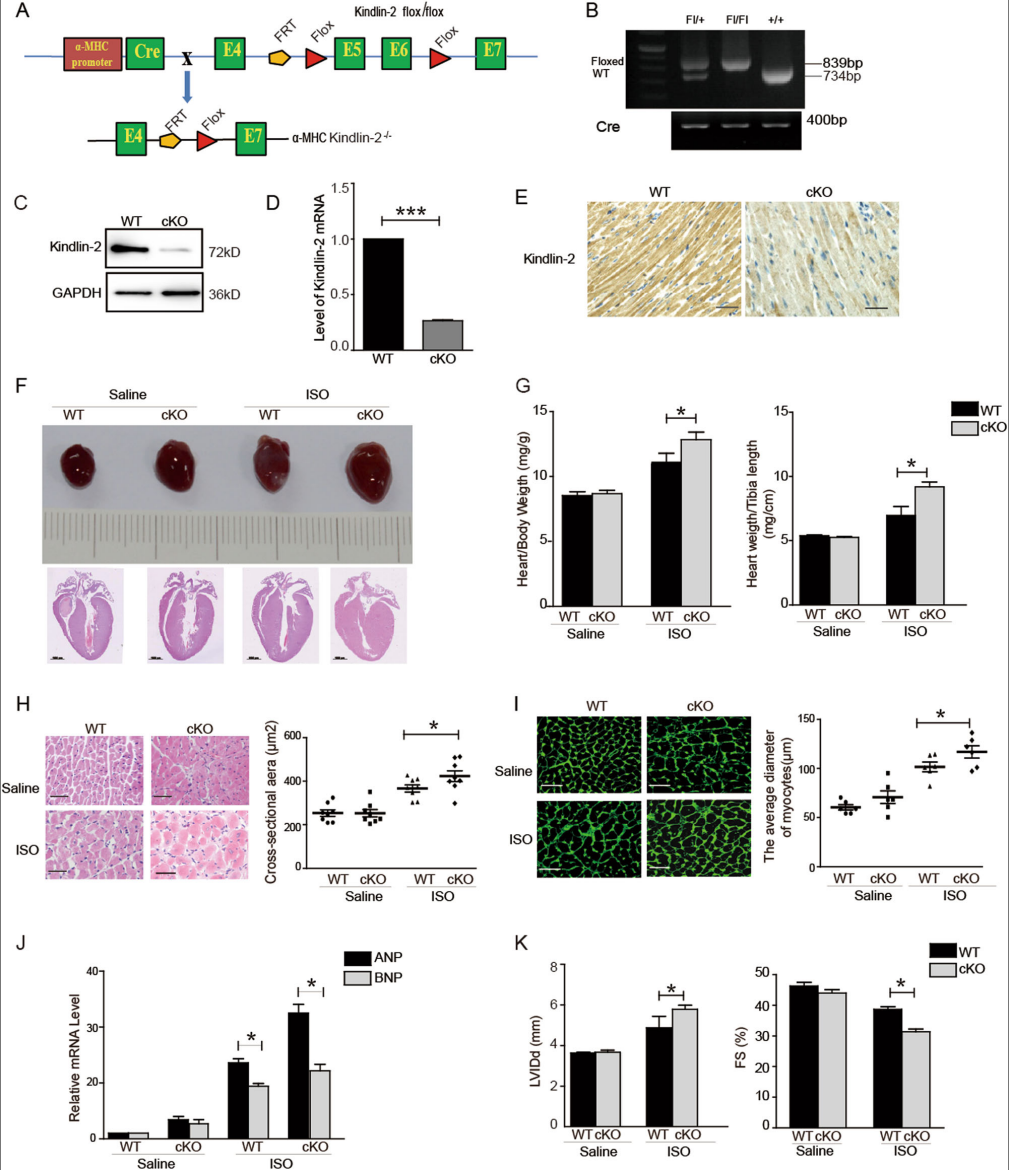
Cardiac-specific Kindlin-2 knockout leads to hypertrophy. **a** Strategy for generating myocardial conditional knockout Kindlin-2 mice. The targeting construct was designed to insert loxP sites flanking exons 5 and 6 of Kindlin-2 gene. Kindlin-2 floxed mice were then crossed with mice expressing recombinant α- MHC-Cre mice to produce Kindlin-2 myocardial conditional knockout (cKO) mice. **b** PCR analysis of extracted genomic DNA from tail clippings. **c**–**e** Western blot, RT-qPCR and immunohistochemical staining showed the level of Kindlin-2 in heart of wild-type or cKO mice (Scale bar = 50 µm). **f** Representative macroscopic observations of hearts (upper panel). HE staining of whole-heart sections (lower panel) (Scale bar = 1000 µm). **g** Ratio of heart weight to body weight or heart weight to tibia length was determined in saline or ISO-treated mice. Means ± S.D. *p < 0.05. **h** HE staining of left ventricular muscle sections (Scale bar = 50 µm) (left panel). The surface areas of cardiomyocyte were quantified (right panel). Means ± S.D. *p < 0.05. **i** Staining of left ventricular muscle sections with cell membrane probe wheat germ agglutinin (Scale bar = 50 µm) (left panel).The average diameter of cardiomyocyte was measured (right panel). Means ± S.D. *P < 0.05. **j** RT-qPCR assays showed mRNA level of *ANP* and *BNP*. **k** The echocardiographic parameters were measured in cKO mice. LVIDd, left ventricular internal diameter (Diastole). FS%, left ventricular shortening fraction. Means ± S.D. *n* = 6 /group. *p < 0.05.

Kindlin-2 cKO mice were generated using the Loxp-Cre system by crossing Kindlin-2fl/flmice with the α-MHC-Cre mice (Fig. 5a). Mice of Kindlin-2 +/+; α-MHC-Cre are referred to as wild-type mice. Mice of Kindlin-2fl/fl; α-MHC-Cre are referred to as cKO mice (Fig. 5b). Kindlin-2 deletion was confirmed by extracting total protein and RNA from hearts of wild-type and Kindlin-2 cKO mice (Fig. 5c, d). Further, immunohistochemical staining indicated that Kindlin-2 was obviously diminished in cardiomyocytes from Kindlin-2 cKO mice (Fig. 5e).

We first compared the morphological difference between 6-month-old Kindlin-2 cKO and wild-type mice and found a marked cardiac enlargement of heart in Kindlin-2 cKO mice (Fig. S4A). The ratio of heart weight to body weight was higher in Kindlin-2 cKO mice than in wild-type mice (Fig. S4B). HE staining revealed the myofibers from Kindlin-2 cKO mice was thicker than those of wild-type mice (Fig. S4C). Further, deletion of Kindlin-2 significantly activated the expression of *ANP* and *BNP* (Fig. S4D). Next, we assess whether cardiac-specific deletion of Kindlin-2 affects cardiac dysfunction. Left ventricular internal diameter (Diastole, LVIDd) of Kindlin-2 cKO mice was increased and ventricular shortening fraction (%FS) of Kindlin-2 cKO mice was decreased, compared with that of wild-type mice (Fig. S4E), indicating that deletion of Kindlin-2 markedly weakens cardiac function. All of these results demonstrated that Kindlin-2 cKO mice developed hypertrophic cardiomyopathy.

Although there were no obvious differences in heart volume between Kindlin-2 cKO mice and wild-type mice at 8 week, ISO treatment with ISO for 1 week obviously enlarged the heart volume of Kindlin-2 cKO mice (Fig. 5f, upper panel). Consistent with macroscopic observations, HE staining showed a marked left ventricular (LV) hypertrophy in ISO-treated Kindlin-2 cKO mice (Fig. 5f, lower panel), suggesting that ISO treatment help Kindlin-2 cKO mice develop hypertrophic cardiomyopathy much earlier. Both ratios of heart weight to body weight and heart weight to the tibia length were increased in ISO-treated Kindlin-2 cKO mice (Fig. 5g). Further, both surface area and diameter of cardiomyocytes in ISO-treated Kindlin-2 cKO mice were larger than that in ISO-treated wild-type mice (Fig. 5h, i). The expression levels of *ANP* and *BNP* were higher in ISO-treated Kindlin-2 cKO mice, compared with ISO-treated wild-type mice (Fig. 5j). Furthermore, ISO treatment further weakened the cardiac function of Kindlin-2 cKO mice (Fig. 5k and Table S2). These results demonstrated that ISO treatment could accelerate the development of hypertrophic cardiomyopathy in Kindlin-2 cKO mice.

### Activation of *GATA4* mediates the hypertrophic cardiomyopathy of Kindlin-2 cKO mice

**Fig. 6:**
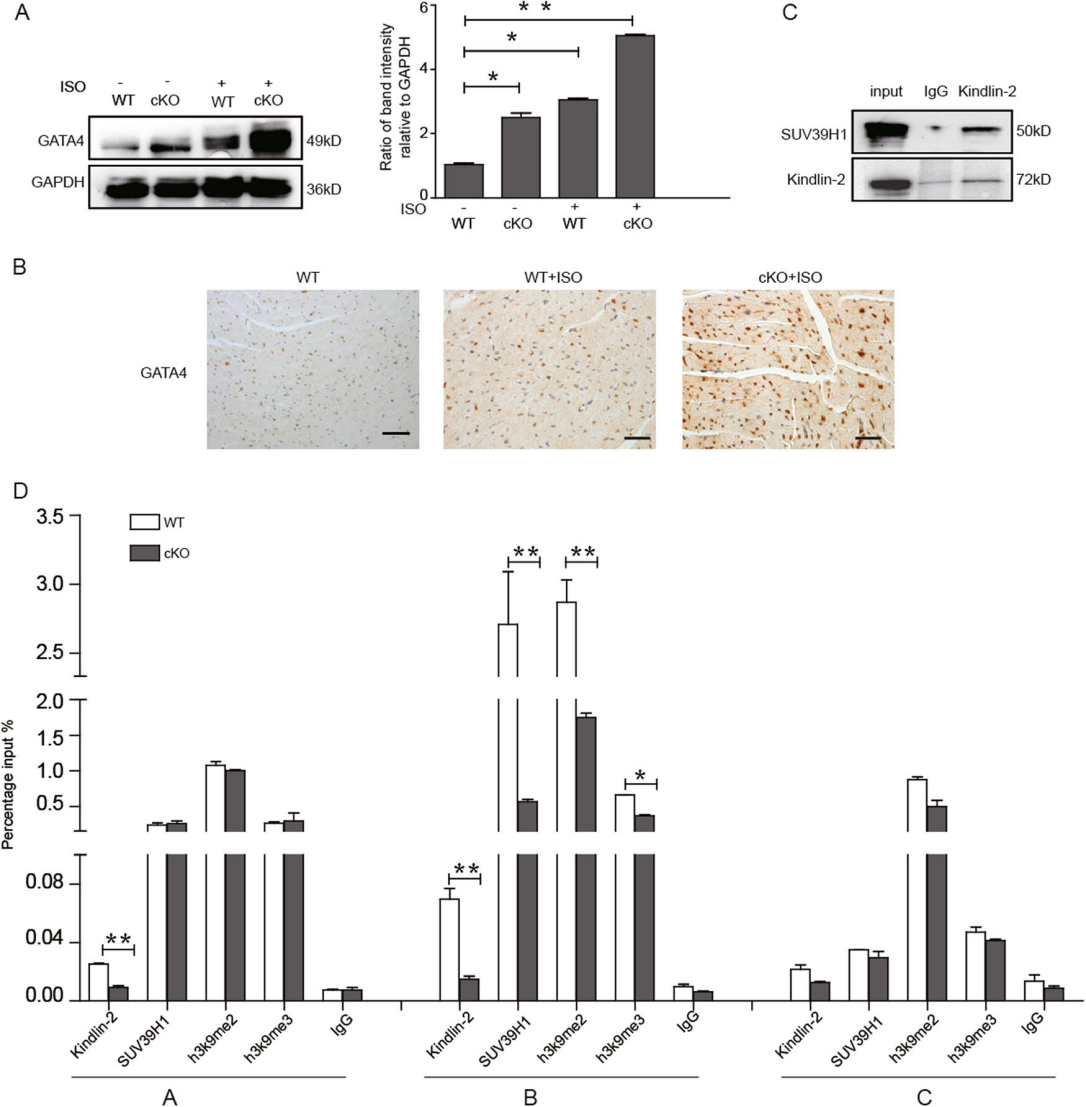
Activation of GATA4 mediates cardiac hypertrophy in Kindlin-2 cKO mice. **a** Wild-type Kindlin-2 or cKO mice were injected with saline or ISO. Total protein was extracted from heart tissue for western blot (left panel). Protein bands on the left were scanned and relative band intensities were normalized (right panel). **b** Immunohistochemical staining showed *GATA4* level (Scale bar = 50 µm). **c** Lysates from heart tissue of wild-type mice were prepared for Co-IP. **d** Lysates were extracted from heart tissue of wild-type and Kindlin-2 cKO mice for ChIP assays. Means ± S.D. *P < 0.05, **p < 0.01.

We isolated cardiac tissues from ISO-treated 8-week-old mice, and results showed that *GATA4* level was significantly enhanced in ISO-treated cKO mice (Fig. 6a, b), suggesting that activation of *GATA4* mediates the hypertrophic cardiomyopathy in Kindlin-2 cKO mice. Next, we performed Co-IP assays to determine the interaction between Kindlin-2 and SUV39H1 in the cardiac tissues of wild-type mice. As shown in Fig. 6c, the interaction between Kindlin-2 and SUV39H1 did occur in the cardiac tissues. Further, we extracted lysates from the cardiac tissues of wild-type mice and Kindlin-2 cKO mice for ChIP assays (Fig. 6d and Fig. S2C). As expected, Kindlin-2, SUV39H1, and SUV39H1-mediated H3K9 methylation were all enriched at the B region of *GATA4* in wild-type mice. However, the enrichments of Kindlin-2, SUV39H1, and H3K9me2 at B region of GATA4 promoter were markedly decreased in Kindlin-2 cKO mice. A modest decrease of H3K9me3 occupancy on B region of GATA4 promoter was also observed in Kindlin-2 cKO mice. These results indicated that activation of GATA4 mediates the hypertrophic cardiomyopathy of Kindlin-2 cKO mice.

**Fig. 7:**
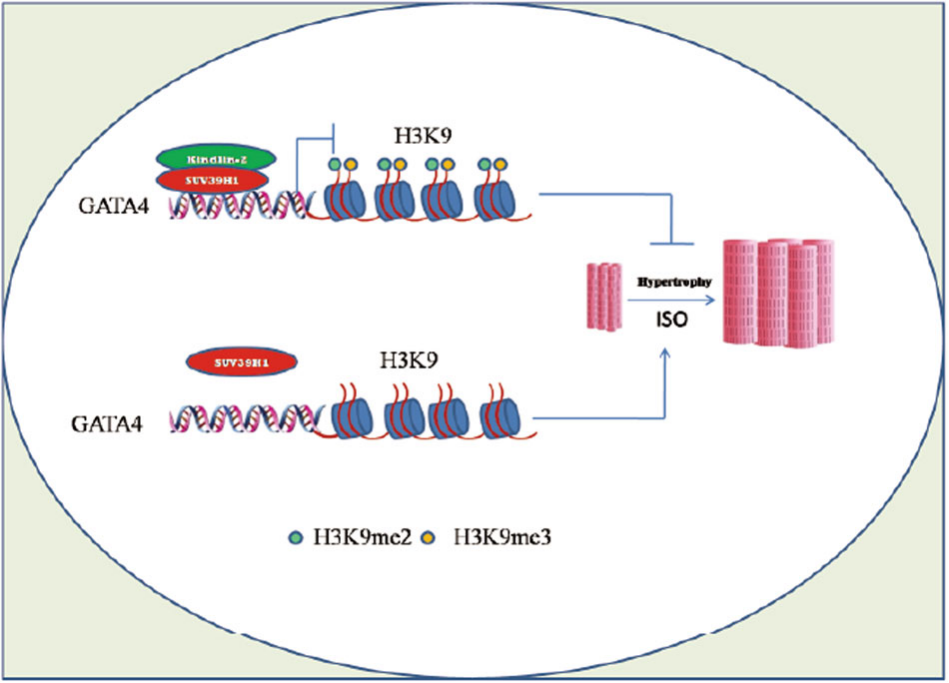
A working model for the role of Kindlin-2 in suppressing GATA4.

Taken together, we suggested that deletion of cardiac Kindlin-2 leads to the dissociation of SUV39H1 from the *GATA4* promoter and in turn H3K9 methylation is erased, leading to the reactivation of *GATA4*. Activation of *GATA4* induces the cardiac hypertrophy (Fig. 7).

## Discussion

Kindlin-2 is a necessary regulator of cardiac structure and function. Depletion of Kindlin-2 results in disruption of Z-disc structures and causes cardiomyopathy and cardiac dysfunction^14^. Targeted deletion of Kindlin-2 in murine heart results in cardiac enlargement of heart and extensive fibrosis of heart. Underlying mechanism is that deletion of Kindlin-2 in heart decreases the protein level of integrin bet^15^, which is focused on the role of Kindlin-2 in cell-to-cell junction. However, not much is known about the role of Kindlin-2 in direct transcriptional regulation of cardiac hypertrophy. In this study, we revealed that Kindlin-2 binds to the promoter of transcription factor *GATA4* and suppresses its transcription, which provides the fundamental evidence for Kindlin-2 in regulating cardiac function.

The transcription factor *GATA4* is a critical regulator of cardiac hypertrophy both in cultured cardiomyocytes and transgenic mice^5^. Loss of *GATA4* attenuates cardiac hypertrophic response and increases myocyote apoptosis^23^. DNA methylation and histone modification have been reported to regulate the expression of *GATA4*. During this process, histone H3K9 acetylation of the *GATA4* promoter is increased, whereas H3K9 dimethylation and DNA methylation are decreased^24^. Mehta et al. reported that MITF governs cardiac hypertrophy by recruiting SWI/SNF to the E box element of GATA4 promoter and inducing H3K4me3 of GATA4, resulting in GATA4 activation^19^. Here, we found that Kindlin-2 modulates GATA4 expression, which may, in part, related to H3K9 methylation of GATA4, but not H3K4me3 (Fig. S5A). In the past, we have reported that Kindlin-2 is involved in DNA methylation regulation of some transcription factors and microRNAs through interacting with DNMT^16,17^. In this study, we revealed that Kindlin-2 interacts with histone methyltransferase SUV39H1 and recruits it to *GATA4* promoter resulting in increased H3K9 methylation of *GATA4* promoter. However, we cannot exclude that Kindlin-2 triggered DNA methylation may also be involved in the suppression of *GATA4*. In fact, histone methylation and DNA methylation often collaborate to regulate the gene expression. It is possible that SUV39H1 and DNMT may be recruited by Kindlin-2 leading to the co-occupancy of H3K9 methylation and DNA methylation at *GATA4* promoter and together suppressing *GATA4* expression. This possibility needs our further investigation.

The family of GATA transcription factors consists of six proteins (GATA-1–6). GATA4 and GATA6 may function in cardiac hypertrophy during adulthood^25^. Similar to GATA4, we also found that loss of Kindlin-2 activates GATA6 expression (Fig. S5B). However, loss of Kindlin-2 has no significant effect on both H3K9 methylation and H3K4 methylation of GATA6, suggesting that both H3K9 methylation and H3K4 methylation are not involved in the regulation of Kindlin-2 on GATA6. Recently, GATA6 is found to be a direct target of miR-203^26^. Our data from a microRNA array indicate that overexpression of Kindlin-2 markedly suppressed miR-203 expression, whereas loss of Kindlin-2 significantly up-regulated the expression of miR-203 (Fig. S5E), suggesting that miR-203 is involved in the regulation of Kindlin-2 on GATA6. Further, the hypertrophic response due to Kindlin-2 knockdown can be blunted by concomitant knockdown of GATA4, but not GATA6. This indicates that GATA6 is not involved into the cardiac hypertrophy induced by Kindlin-2.

## Supplementary information

Figure S1

Figure S2

Figure S3

Figure S4

Figure S5

Table S1

Table S2

supplemental figure legends

DECLARATION OF CONTRIBUTIONS TO ARTICLE
