# Supplementary figures and images for: Kindlin-2 suppresses transcription factor GATA4 through interaction with SUV39H1 to attenuate hypertrophy
Source: Cell Death Dis. 2019 Nov 26;10(12):890. doi: 10.1038/s41419-019-2121-0 (PMC6877536; doi:10.1038/s41419-019-2121-0)

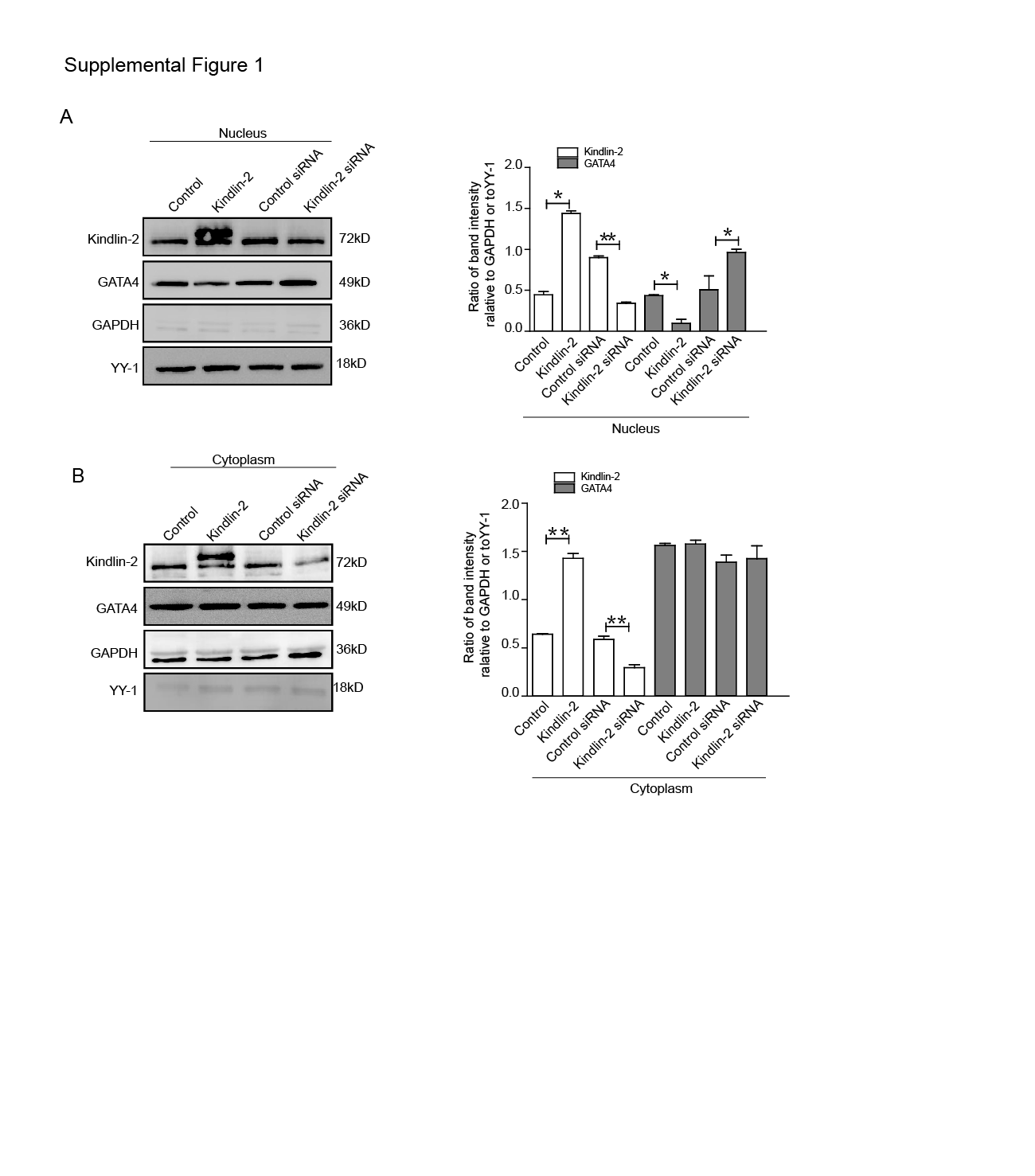

Supplement: Supplementary file 1 — Figure S1 [file 41419_2019_2121_MOESM1_ESM.tif]

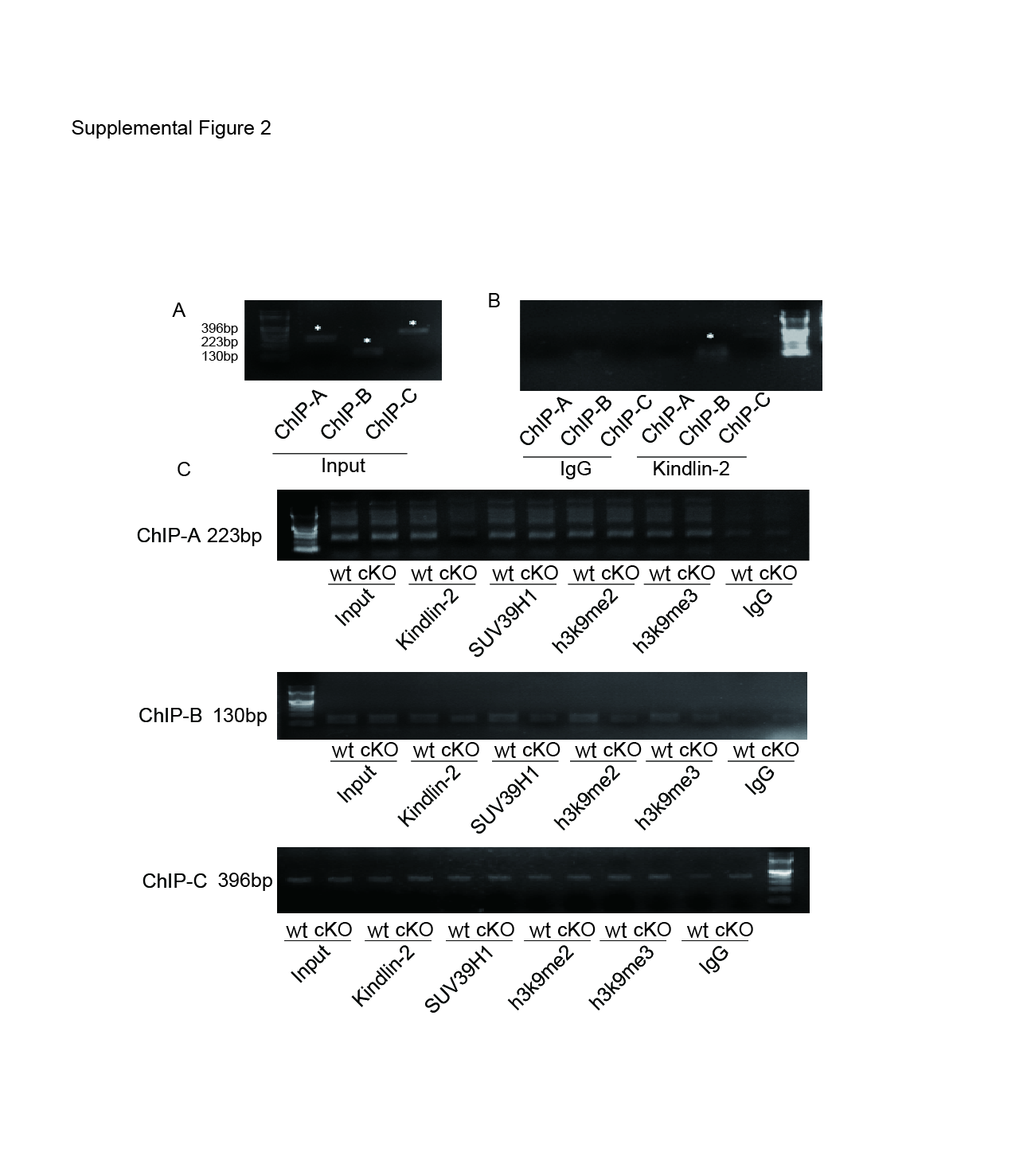

Supplement: Supplementary file 2 — Figure S2 [file 41419_2019_2121_MOESM2_ESM.tif]

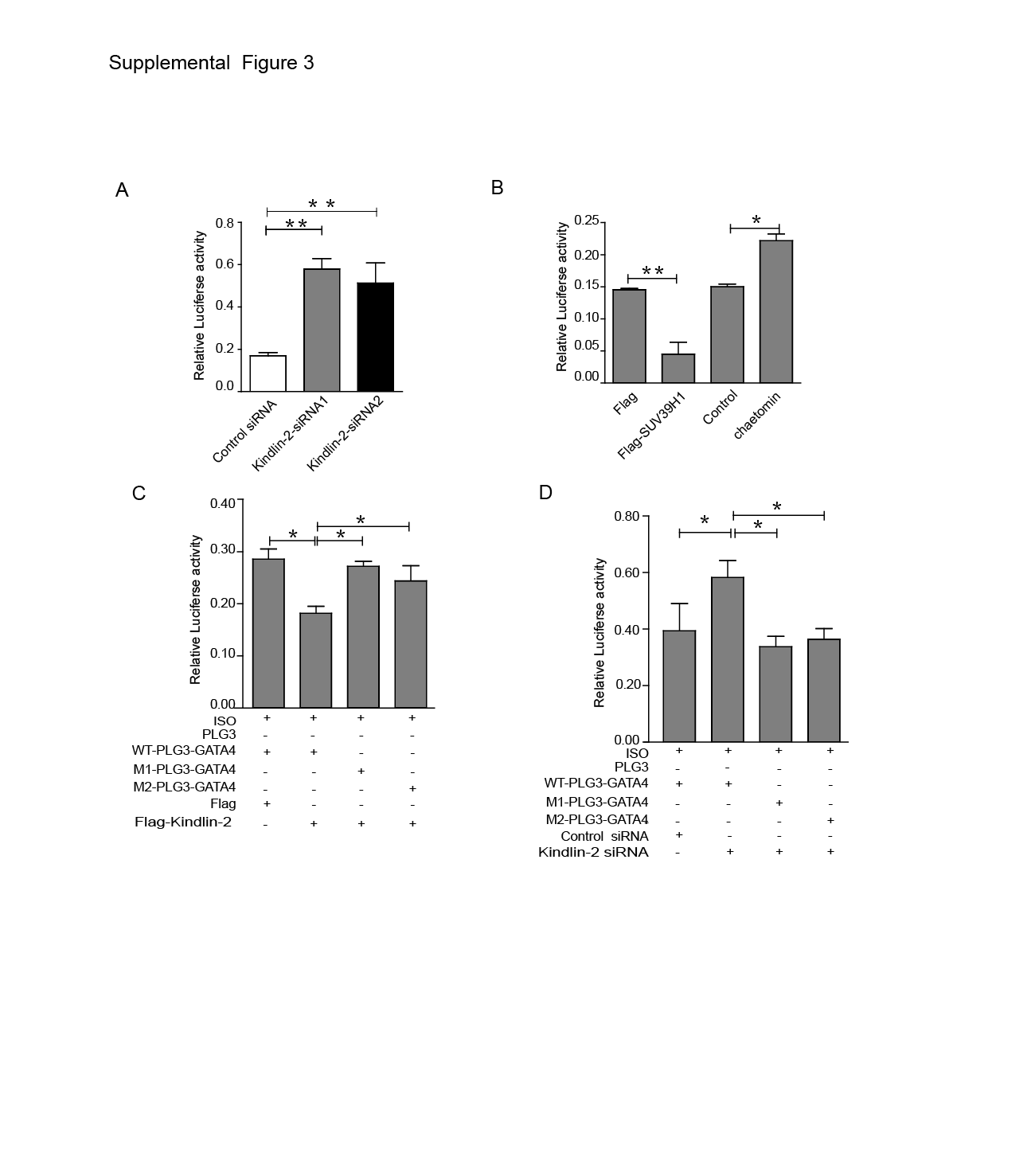

Supplement: Supplementary file 3 — Figure S3 [file 41419_2019_2121_MOESM3_ESM.tif]

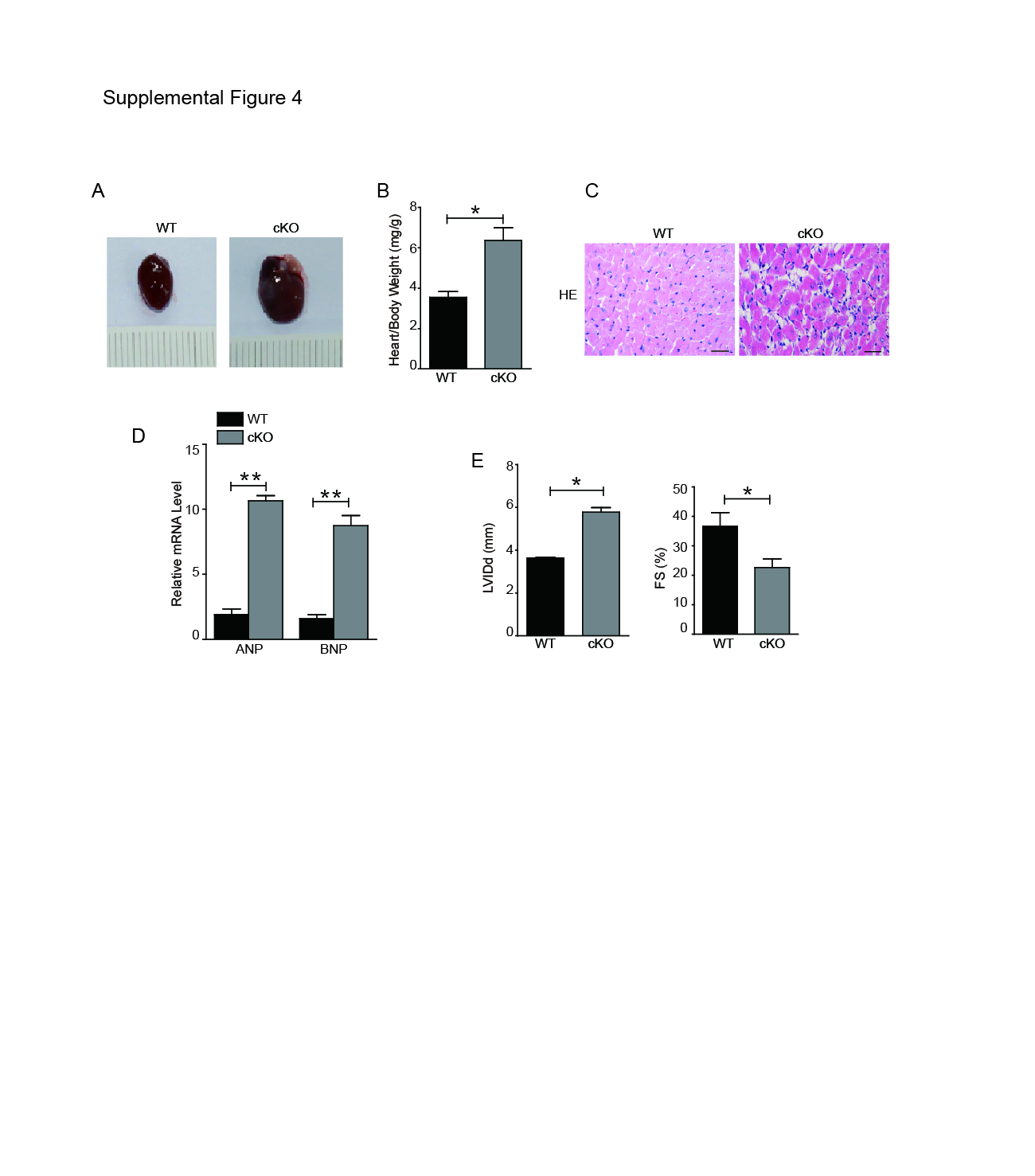

Supplement: Supplementary file 4 — Figure S4 [file 41419_2019_2121_MOESM4_ESM.tif]

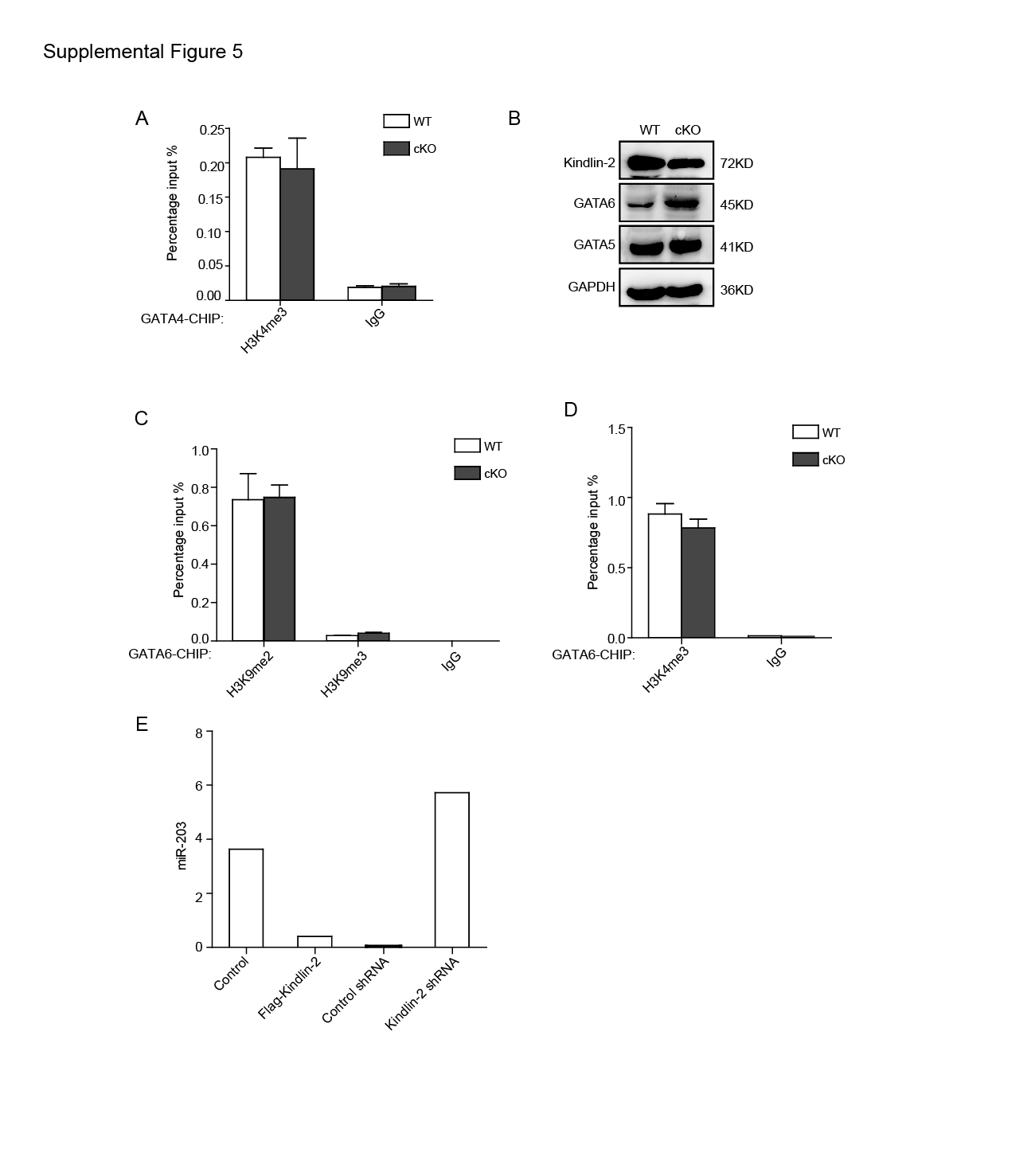

Supplement: Supplementary file 5 — Figure S5 [file 41419_2019_2121_MOESM5_ESM.tif]
