# Supplementary material for: Kindlin-2 suppresses transcription factor GATA4 through interaction with SUV39H1 to attenuate hypertrophy
Source: Cell Death Dis. 2019 Nov 26;10(12):890. doi: 10.1038/s41419-019-2121-0 (PMC6877536; doi:10.1038/s41419-019-2121-0)
Supplement: Supplementary file 8 — supplemental figure legends [file 41419_2019_2121_MOESM8_ESM.docx]

Figure. S1. Both nuclear (A) and cytoplasmic (B) lysates were extracted from Kindlin-2-overexpressing or Kindlin-2-depleted cardiomyocytes for western blotting analysis using the indicated antibodies. The absence of GAPDH indicated the fraction was from the nucleus. The absence of YY1 indicates that the fraction was from the cytoplasm. Relative band intensity of western blotting was analyzed.

Figure. S2. (A) Agarose gel electrophoresis showed that the three primer pairs have relatively similar amplification efficacy. (B) Lysates were extracted from mouse cardiac tissues for ChIP assays using anti-Kindlin-2 antibody. Agarose gel electrophoresis was then performed using the three primers of GATA4. (C) Lysates were extracted from heart tissue of wild-type mice and Kindlin-2 cKO mice for ChIP assays using the indicated antibodies. Agarose gel electrophoresis was then performed using the three primers of GATA4.

Figure. S3. (A) Luciferase reporter assays were performed in Kindlin-2-depleted cardiomyocytes. The values were normalized to Renilla luciferase. (B) HEK293A cells were transfected with Flag-SUV39H1 or treated by Chaetomin and luciferase reporter activity of GATA4 promoter was measured. (C) HEK293A cells were co-transfected with the indicated plasmids with or without ISO treatment. Luciferase reporter activity was measured. (D) HEK293A cells were co-transfected with the indicated plasmids and siRNAs with or without ISO treatment. Luciferase reporter activity was measured. Data are mean ± S.D. * indicates p<0.05, ** indicates p<0.01 by Student’s *t-*test.

Figure. S4. (A) Representative macroscopic observation of hearts from the wild-type mice and Kindlin-2cKO mice at postnatal 6 month postnatal. (B) The ratio of heart weight to body weight was determined in wild-type mice and Kindlin-2 cKO mice. Date are shown as means ± S.D. **, p<0.01 by Student’s *t -*test. (C) Heart sections from wild-type and Kindlin-2 cKO mice were stained with HE (Scale bar=50µm). (D) Total RNA was extracted from heart tissue of wild-type and Kindlin-2 cKO mice for RT-qPCR assay to detect the mRNA level of *ANP* and *BNP*. (E) Echocardiographic parameters were measured in wild-type and Kindlin-2cKO mice. LVIDd, left ventricular internal diameter (Diastole). FS﹪, left ventricular shortening fraction. Values are means ± S.D. from 6 mice of each group. * indicates P<0.05 by Student’s *t-*test.

Figure. S5. (A) Lysates were extracted from heart tissue of wild-type mice and Kindlin-2 cKO mice for ChIP assays using anti-H3K4me3 antibodies. RT-qPCR was then performed to quantify ChIP-enriched DNAs using GATA4 primer B. (B) Western blot analysis in wild-type mice and Kindlin-2 cKO mice using the indicated antibodies. (C-D) Lysates were extracted from heart tissue of wild-type mice and Kindlin-2 cKO mice for ChIP assays using anti-H3K9me2, anti-H3K9me3 and anti-H3K4me3 antibodies. RT-qPCR was then performed to quantify ChIP-enriched DNAs using GATA6 primer. (E) The level of miR-203 in Kindlin-2 overexpressing cells and Kindlin-2 knockdown cells.

Table S1. RNA sequencing was performed in control or Kindlin-2 siRNA-treated primary neonate rat cardiomyocytes. Up-regulated genes in RNA sequencing data were shown.

Table S2. Echocardiographic Data of WT and Kindlin-2 cKO Mice.
