# Supplementary material for: Kindlin-2 suppresses transcription factor GATA4 through interaction with SUV39H1 to attenuate hypertrophy
Source: Cell Death Dis. 2019 Nov 26;10(12):890. doi: 10.1038/s41419-019-2121-0 (PMC6877536; doi:10.1038/s41419-019-2121-0)
Supplement: Supplementary file 9 — DECLARATION OF CONTRIBUTIONS TO ARTICLE [file 41419_2019_2121_MOESM9_ESM.pdf]

DECLARATION OF CONTRIBUTIONS TO ARTICLE

ADMC

Manuscript Number: CDDIS-19-1319RR

Journal Name: *Cell Death & Disease* (the 'Journal')

Proposed Title of the Contribution:

Kindlin-2 suppresses transcription factor GATA4 through interaction with SUV39H1 to attenuate hypertrophy (the 'Contribution)

Author(s):

Lihua Qi, Xiaochun Chi, Xi Zhang, Xueqian Feng, Wenhui Chu, Shengchang Zhang, Junzhou Wu, Yao Song, Youyi Zhang, Wei Kong, Yu Yu, Hongquan Zhang (the 'Authors')

For all *CDDis* articles, each person named as an author in the published version must be able to show he or she has contributed substantially to the article.

Authorship credit should be based on 1) substantial contributions to conception and design, acquisition of data, or analysis and interpretation of data; 2) drafting the article or revising it critically for important intellectual content; and 3) final approval of the version to be published. Authors should meet conditions 1, 2 and 3.

Any person who cannot be shown to have made a substantial contribution to the article cannot be listed as an author in the final version. The name of any person who is deemed to have made a minor contribution can, however, appear in the Acknowledgments section of the article.

Please complete the table below to indicate the contributions of all named authors to the manuscript.

Author Full Name:	Specification of Contribution to the Manuscript:
Lihua Qi	Designed the project, performed most of the experiments, analyzed the data, wrote the manuscript.
Xiaochun Chi	Construct cardiac muscle specific Kindlin-2 KO mice, wrote the related Methods section.
Xi Zhang	Identified the Kindlin-2 KO mice, performed ChIP and agarose gel electrophoresis, analyzed the related data.
Xueqian Feng	Performed Western blot analysis, analyzed the related data, wrote the related Methods section.
Wenhui Chu	Performed RNA seq and qPCR analysis, analyzed the related data, wrote the related Methods section.
Shengchang Zhang	Performed immunostaining analysis, wrote the related Methods section.
Junzhou Wu	Constructed all the plasmids, expressed and purified GST-SUV39H1 protein, wrote the related Methods section.
Yao Song	Performed echocardiographic analysis, analyzed the related data.
Youyi Zhang	Analyzed the echocardiographic data, revised the related content.
Wei Kong	Guided animal model construction, analyzed the related data,revised the related content.
Yu Yu	Designed the project, analyzed and interpreted the data, wrote the manuscript.
Hongquan Zhang	Designed the project, analyzed and interpreted the data, revised the manuscript.

Please complete the table below to indicate the contributions of all named authors to the figures.

Figure 1:

Lihua Qi, Wenhui Chu, Xi Zhang, Yu Yu, Hongquan Zhang

Figure 2:

Lihua Qi, Wenhui Chu, Yu Yu, Hongquan Zhang

Figure 3:

Lihua Qi, Junzhou Wu, Yu Yu, Hongquan Zhang

Figure 4:

Lihua Qi, Xueqian Feng, Wei Kong, Yu Yu, Hongquan Zhang

Figure 5:

Lihua Qi, Xiaochun Chi, Xi Zhang, Shengchang Zhang, Yao Song, Youyi Zhang, Yu Yu, Hongquan Zhang

Figure 6:

Lihua Qi, Xi Zhang, Xueqian Feng, Yu Yu, Hongquan Zhang

Signed for and on behalf of the Author(s):	Print Name:	Date:
[signature]	Hongquan Zhang	2019-10-14
